# Melanocortin 1 Receptor: Structure, Function, and Regulation

**DOI:** 10.3389/fgene.2016.00095

**Published:** 2016-05-31

**Authors:** Erin M. Wolf Horrell, Mary C. Boulanger, John A. D’Orazio

**Affiliations:** ^1^Department of Physiology, University of Kentucky College of MedicineLexington, KY, USA; ^2^Markey Cancer Center, University of Kentucky College of MedicineLexington, KY, USA; ^3^Departments of Pediatrics, Toxicology and Cancer Biology, Physiology, and Pharmacology and Nutritional Sciences, University of Kentucky College of MedicineLexington, KY, USA

**Keywords:** melanocyte, melanoma, MC1R, melanocortin, ASIP, βD3, ATR, DNA repair

## Abstract

The melanocortin 1 receptor (MC1R) is a melanocytic G_s_ protein coupled receptor that regulates skin pigmentation, UV responses, and melanoma risk. It is a highly polymorphic gene, and loss of function correlates with a fair, UV-sensitive, and melanoma-prone phenotype due to defective epidermal melanization and sub-optimal DNA repair. MC1R signaling, achieved through adenylyl cyclase activation and generation of the second messenger cAMP, is hormonally controlled by the positive agonist melanocortin, the negative agonist agouti signaling protein, and the neutral antagonist β-defensin 3. Activation of cAMP signaling up-regulates melanin production and deposition in the epidermis which functions to limit UV penetration into the skin and enhances nucleotide excision repair (NER), the genomic stability pathway responsible for clearing UV photolesions from DNA to avoid mutagenesis. Herein we review MC1R structure and function and summarize our laboratory’s findings on the molecular mechanisms by which MC1R signaling impacts NER.

## Melanocortin Receptors

The melanocortin (MC) receptor family is the smallest member of the class A (rhodopsin-like) family of G-protein coupled receptors (GPCRs) (Gether, 2000; Montero-Melendez, 2015) and consists of five members: MC1R, MC2R, MC3R, MC4R, and MC5R with varying tissue expression and functions. MC1R is found on both melanocytes and leukocytes and its activation promotes UV resistance and anti-inflammatory signaling, respectively, (Mountjoy et al., 1992). MC2R, cloned Mountjoy et al. (1992), is found in the adrenal cortex. MC3R, cloned Desarnaud et al. (1994), and MC4R, cloned Gantz et al. (1993), are both found primarily in the CNS regulating food intake and sexual function. MC5R, located in skeletal muscle and brain, has an exocrine function (Gantz et al., 1994a). Sequence homology between the five receptors is only 40–60% which accounts for the lack of ligand specificity between receptors (Gantz et al., 1993; Yang et al., 2003). This review will focus on the role of MC1R in melanocytes with an emphasis on ligands, signaling pathways, structure, and function.

## Melanocortin 1 Receptor (MC1R)

The human MC1R is 317 amino acids (Garcia-Borron et al., 2005), and it was originally identified and cloned by two independent groups Chhajlani and Wikberg (1992) and Mountjoy et al. (1992) and mapped to chromosome 16q24.3 Gantz et al. (1994b). The receptor is primarily located on melanocytes and transformed melanoma cells (Ghanem et al., 1988; Siegrist et al., 1989, 1994; Donatien et al., 1992). MC1R protein expression is typically low, with an estimated 700 protein units expressed per melanocyte and somewhat higher numbers on melanoma cells (Donatien et al., 1992; Roberts et al., 2006). The 315 amino acid murine homolog, Mc1r, was also cloned and identified Mountjoy et al. (1992) and mapped to the *extension* locus Robbins et al. (1993). Mice with a mutated *extension* locus display a reddish blonde coat color instead of the darkly pigmented black coat color typically found on the C57BL/6 background, thus providing the first genetic evidence that MC1R may play an important role in the regulation of pigment (Searle, 1968).

### MC1R Structure

**FIGURE 1:**
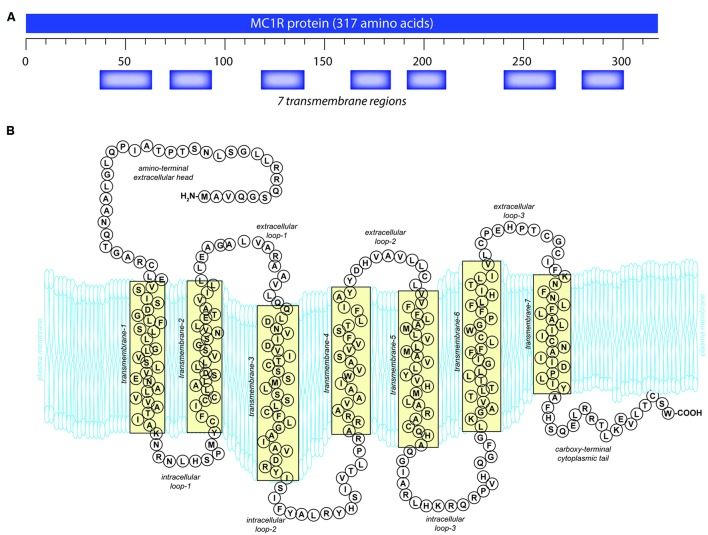
**Melanocortin 1 receptor (MC1R) gene and protein structures. (A)** The human MC1R locus (cytogenetic location: 16q24.3) encodes a seven transmembrane protein that is highly polymorphic. **(B)** The mature MC1R protein is a G_s_-protein coupled receptor (GPCR) that spans the membrane seven times. Extracellular and transmembrane domains engage MC1R ligands while intracellular and transmembrane domains regulate adenylyl cyclase interactions and signaling.

Like other GPCRs, MC1R is made up of 7 α-helical transmembrane (TM) domains with a DRY motif at the junction of the third TM domain, an intracellular C-terminus with a palmitoylation site, and an extracellular N-terminus with an N-linked glycosylation site (Yang, 2011) (**Figure 1**). Unique to the MC receptor subfamily compared to other GPCRs is the lack of one or two cysteines in the first and second extracellular domains and lack of proline in the fourth and fifth TM domains (Yang, 2011).

#### N-terminus and c-terminus

The extracellular N-terminal tail (**Figure 1**) functions both for ligand affinity (Chhajlani et al., 1996) and as a signal anchor (Wallin and von Heijne, 1995; Garcia-Borron et al., 2005). There is a conserved cysteine residue located at the junction of the N-terminus and the first TM domain which is absolutely required for receptor function (Frandberg et al., 2001; Sanchez-Laorden et al., 2006b). The C-terminus in GPCR often plays a role in protein trafficking from the endoplasmic reticulum to the plasma membrane (Schulein et al., 1998; Qanbar and Bouvier, 2003) and also in receptor interactions with the G protein at the plasma membrane (Strader et al., 1994). Similar to the other MC receptors, MC1R has a characteristically short C-terminal tail that is only 14 amino acids in length (**Figure 1**). A pentapeptide present on the C-terminal tail contains the invariant tripeptide sequence T314, C315, and W317 present in all MC receptors. The pentapeptide, and specifically the invariant tripeptide, are required for translocation of the receptor to the plasma membrane (Sanchez-Mas et al., 2005b). Mutations which disrupt the pentapeptide or specifically the invariant tripeptide such as premature termination at R306 (Newton et al., 2000) or deletion of the terminal pentapeptide (Sanchez-Mas et al., 2005b), result in decreased plasma membrane MC1R expression. Although C-terminal deletions have detrimental effects, additional amino acids on the end of the C-terminus do not appear to affect MC1R function. A splice variant exists with an additional 65 amino acids that displays similar pharmacology to the unspliced protein (Tan et al., 1999). In addition to affecting receptor localization to the plasma membrane, the C-terminus also plays a role in desensitization and internalization (Pitcher et al., 1998; Luttrell and Lefkowitz, 2002).

#### Intracellular and Extracellular Loops

Melanocortin 1 receptor’s intracellular and extracellular loops (ils and els, respectively) are found between the transmembrane regions (**Figure 1**) and have conserved sequences found across many MC receptors. MC1R els are small compared to most GPCRs but are critical for basal constitutive signaling activity (Holst and Schwartz, 2003). Because the els of MC1R interact with ligands, mutations in this region impact binding affinity (Chhajlani et al., 1996). El3 in particular appears to play a critical role in melanocortin affinity through conserved proline and cysteine residues (Holst and Schwartz, 2003). El3 interacts with TM6 and TM7 which are also required for ligand-receptor binding, and it is believed that C267 and C275 in el3 form disulfide bonds between TM6 and TM7 affecting the tertiary structure of the receptor (Frandberg et al., 2001; Holst and Schwartz, 2003; Garcia-Borron et al., 2005).

Similarly, MC1R ils are important for binding of the G_s_ protein and have sites for phosphorylation that affect signal regulation, internalization, and receptor cycling (Strader et al., 1994). Il1 is important for normal activation, and mutations of this domain increase MC1R signaling activity as seen in the *tobacco* mutation (S69L in mouse; S71 in human). Six mutations have been reported in il1, four of which cause a loss of signaling function (Robbins et al., 1993). As is characteristic for class A GPCRs, the tripeptide 141 DRY 143 located at the interface of il2 and TM3 is required for MC1R function (Schioth et al., 1999). Additionally, there are putative protein kinase A (PKA) and protein kinase C phosphorylation sites in il2 of both human and mouse (and il3 in mouse), however, neither PKA nor PKC has been shown to phosphorylate MC1R to date (Garcia-Borron et al., 2005).

### MC1R Oligomerization

Like many other GPCRs, oligomerization of MC1R is functionally important for modulation of ligand binding, coupling efficiency, desensitization, and trafficking through the endoplasmic reticulum (Sanchez-Laorden et al., 2006a). MC1R undergoes constitutive dimerization without a ligand binding requirement (Mandrika et al., 2005; Sanchez-Laorden et al., 2006a) at the level of the ER (Sanchez-Laorden et al., 2006a). MC1R homo-dimerization is dependent upon both covalent and non-covalent interactions rather than a coiled-coil mechanism, mediated by four inter-subunit disulfide bonds at C35, C267, C273, and C275 or by domain swapping. Disruption of any disulfide bond abolishes MC1R function, however, only C35 is required for MC1R to travel from the ER to the plasma membrane. Although mutation of C35 prevents translocation of MC1R to the plasma membrane, the protein can still dimerize, therefore dimerization is not sufficient for ER to plasma membrane transport (Sanchez-Laorden et al., 2006a).

Dimerization of heterogeneous receptors can have a dramatic effect on MC1R signaling. Dimerization between mutant and wild-type MC1R proteins can cause a dominant negative effect (Sanchez-Laorden et al., 2006a) similar to that reported in dimerization of mutant MC4R with wild-type MC4R (Biebermann et al., 2003). Similarly, dimerization between wild type and a mutant MC1R unable to translocate to the surface of the cell also resulted in dose-dependent dominant negative inhibition of wild-type MC1R cell surface localization (Sanchez-Laorden et al., 2006a). Conversely, coupling with wild type GPCR can partially rescue function of mutant GPCR via exchange of defective domains (Breitwieser, 2004). Co-expression of two MC1R mutants with mutations in different domains may similarly rescue function through complementation, however, rescue was not observed if mutations were in the same domain (Sanchez-Laorden et al., 2006a). MC1R dimerization characteristics could therefore impact melanocortin signaling and play an important role in individuals with inherited polymorphisms in the MC1R protein.

### MC1R Desensitization

Like other GPCRs, MC1R desensitization and internalization represent major mechanisms whereby its function can be regulated (Pitcher et al., 1998). Multiple members of the melanocortin receptor family undergo homologous desensitization including murine Mc2R (Baig et al., 2001), murine Mc4r (Shinyama et al., 2003), and both murine Mc1R and human MC1R (Sanchez-Mas et al., 2005a). MC1R undergoes homologous desensitization following short exposure to its positive agonist, α-MSH in a PKA independent and G protein coupled receptor kinase (GRK) dependent manner. Following agonist stimulation, GRKs phosphorylate GPCRs resulting in receptor decoupling from the G protein and subsequent internalization (Benovic et al., 1985; Hausdorff et al., 1990). MC1R desensitization is dependent upon GRK2 and GRK6, however, internalization only requires GRK6 phosphorylation of T308 and S316 on the C-terminus (Sanchez-Laorden et al., 2007). Studies conducted in primary human melanocytes, however, demonstrated that GRK expression varies between individuals (Swope et al., 2012). In addition, MC1R desensitization is further mediated via β-arrestins (ARRB). ARRBs bind the phosphorylated receptor and prevent the receptor from coupling to the G protein and target the receptor for internalization (Attramadal et al., 1992). Recently, ARRB2 but not ARRB1 has been shown to play a role in receptor desensitization and internalization (Abrisqueta et al., 2013).

### Melanocortin Signaling and cAMP

**FIGURE 2:**
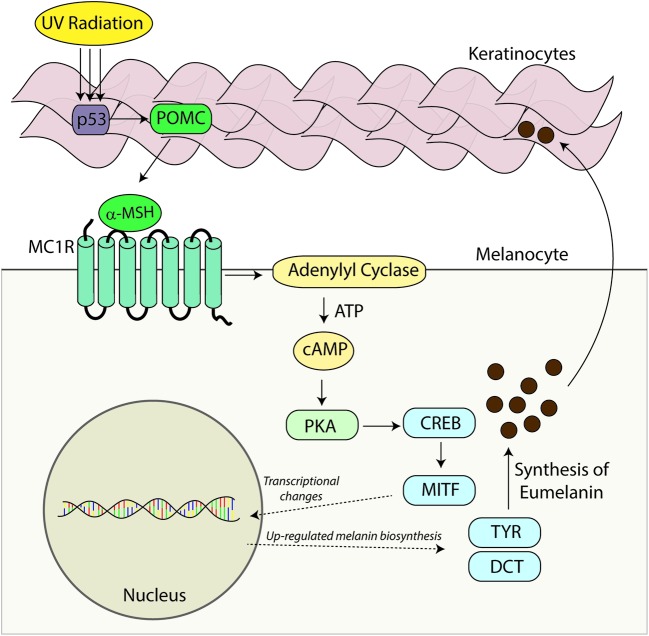
**Melanocortin – MC1R signaling axis.** Melanocortins (α-MSH, ACTH) are produced basally by the pituitary and induced in the skin after UV injury. Binding of these melanocortin ligands to MC1R promotes critical UV-resistance physiologic changes in melanocytes and protects the skin from UV damage. Upon binding melanocortins, MC1R activates adenylyl cyclase and stimulates cAMP production. In turn, a variety of downstream effector pathways including induction of the CREB and Mitf transcription factor networks and an increase in the activity of PKA takes place. Expression of a variety of enzymes including tyrosinase (TYR) and dopachrome tautomerase (DCT) involved in melanin biosynthesis is increased and melanin production is up-regulated. Melanin produced in organelles termed melanosomes, is transferred to neighboring keratinocytes and in this way a UV-protective layer of pigment in the epidermis is established to enhance the skin’s ability to resist further UV injury. Importantly, melanocytic genomic stability is also enhanced through improved DNA repair. In the absence of a functional melanocortin signaling axis, these pathways are blunted, the skin is under-melanized and melanocytes accumulate more UV mutations as a result of ineffective DNA repair. In this way, individuals with inherited defects in MC1R signaling are at heightened risk for melanoma.

Melanocortin 1 receptor is complexed to the heterotrimeric G protein. Following activation with agonistic ligands the Gα_s_ protein dissociates from MC1R and stimulates adenylyl cyclase activity which cleaves ATP to generate the second messenger cAMP (**Figure 2**). In melanocytes, increased cAMP levels lead to a host of downstream signaling events including activation of effector proteins such as cAMP-dependent protein kinase (PKA) (Neves et al., 2002; Dorsam and Gutkind, 2007). In this manner MC1R signaling activates various signaling cascades within the cell. In melanocytes, cAMP induction leads to increased melanin synthesis (Suzuki et al., 1997; Abdel-Malek et al., 2014) and resistance to UV injury through enhanced antioxidant defenses and acceleration of nucleotide excision repair (NER) (Kokot et al., 2009; Song et al., 2009; Kadekaro et al., 2010, 2012; Abdel-Malek et al., 2014; Jarrett et al., 2014). The dissociated Gαβ protein can also modify intracellular signaling including the mitogen-activated protein kinase family which affects a multitude of signaling pathways (further reviewed by Dorsam and Gutkind, 2007). MC1R, like other GPCRs, displays some degree of ligand independent basal signaling (Chalmers and Behan, 2002; Milligan, 2003). This has been demonstrated for both human MC1R (Mas et al., 2003) and murine Mc1r (Jackson et al., 2007), human MC3R, human MC4R, and murine Mc5r (Nijenhuis et al., 2001). Genetic proof of basal MC1R signaling is evident in *Pomc1* knockout mice which are incapable of generating melanocortins which are the major agonists of MC1R. In contrast to *Mc1r*-defective strains such as *extension*, POMC-null mice maintain a dark coat color, suggesting Mc1r has constitutive ligand independent activity (Bennett and Lamoreux, 2003).

#### MC1R and Pigmentation

There are two major types of pigment present in the skin, the darkly pigmented eumelanin and the red/yellow sulfated pheomelanin. Eumelanin is chemically inert and is highly photoprotective by absorbing UV radiation (Kaidbey et al., 1979; Scherer and Kumar, 2010) and oxidants (Hoogduijn et al., 2004). In contrast, pheomelanin is much less efficient at blocking penetration of UV radiation into the skin and can promote UV-induced cellular damage by contributing to free radical and oxidative injury (Thody et al., 1991; Mitra et al., 2012). MC1R signaling is a major determinant for the amount and type of melanin pigments synthesized by melanocytes, regulating both basal pigmentation and the UV induced tanning response (D’Orazio et al., 2006). MC1R signaling increases eumelanin synthesis, the ratio of eumelanin-to-pheomelanin (Hunt et al., 1995), and enhances melanosome transfer to enhance melanin deposition in keratinocytes (Virador et al., 2002).

**FIGURE 3:**
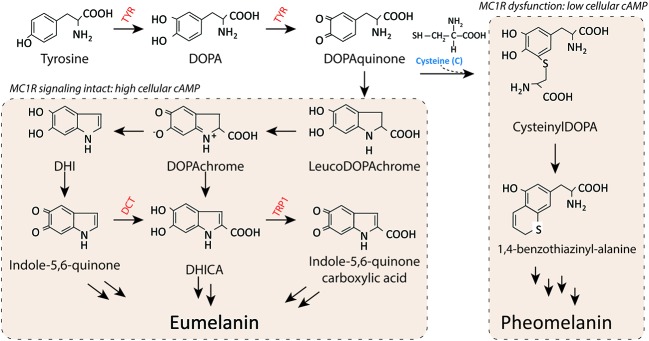
**Melanin synthesis.** There are two main types of melanin: the dark brown/black UV-protective eumelanin and the red/blonde sulfated pheomelanin pigment. Each is derived from progressive cyclization and oxidation of the amino acid tyrosine. Tyrosinase, the rate-limiting enzyme for melanogenesis, catalyzes the first two stages of melanin biosynthesis. When MC1R is functional and cAMP levels are high, melanocytes produce eumelanin preferentially. In contrast, when MC1R is dysfunctional and cAMP levels are low, cysteine is incorporated and pheomelanin is made instead. Important pigment enzymes which cause hypopigmentary phenotypes when defective include tyrosinase (TYR), dopachrome tautomerase (DCT), and tyrosinase related protein-1 (TRP1). Complexion and UV resistance are mainly determined by the amount of eumelanin in the skin.

Both eumelanin and pheomelanin derive from the sequential cyclization and oxidation of the amino acid tyrosine (**Figure 3**) (Ito, 2003). The first two biosynthetic steps are shared between the two pathways: the conversion of tyrosine to DOPA and then to DOPAquinone by the enzyme tyrosinase. Eumelanogenesis and pheomelanogenesis diverge after formation of DOPAquinone. Other enzymes beside tyrosinase are needed for melanin synthesis including dopachrome tautomerase and tyrosinase-related protein 1. Defects in many pigment enzymes yield hypomelanotic phenotypes such as albinism (Baxter and Pavan, 2013). Pheomelanin production is dependent upon the incorporation of a cysteine and retention of sulfur after the synthesis of DOPAquinone, which may explain why mature pheomelanin pigments are reddish/yellow rather than dark brown/black as eumelanin is. Although the control of the pigment switch between eumelanin and pheomelanin is regulated by multiple factors including the pH of the cellular milieu and the levels of tyrosinase (Burchill and Thody, 1986; Ancans et al., 2001), the presence of a functional MC1R is required for effective synthesis of eumelanin. Since eumelanin absorbs UV radiation, the more eumelanin the skin has, the more protected it is from UV damage.

#### MC1R Variants

**FIGURE 4:**
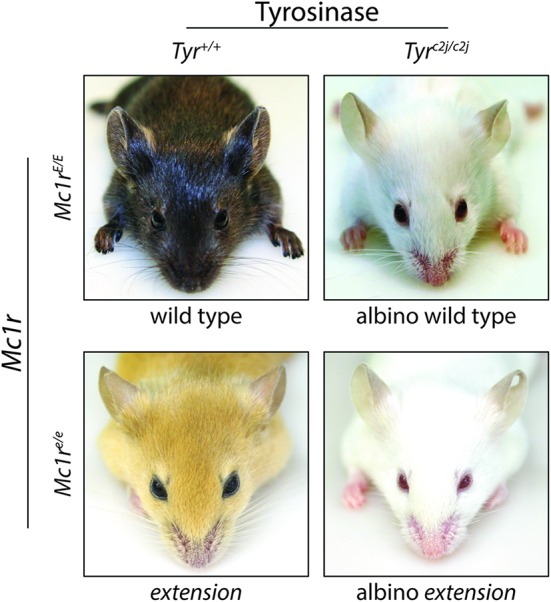
**Melanocortin 1 receptor controls coat color in C57BL/6 mice.** C57BL/6 mice with wild type (functional) *Mc1r* exhibit a eumelanotic phenotype, whereas congenic animals defective for Mc1r, such as *extension* mice, exhibit a pheomelanotic coat color. Shown are *Mc1r^E/E^* (wt) and *Mc1r^e/e^ (extension)* age-matched animals along with C57BL/6 *Tyr^c2j/c2j^* animals in either the *Mc1r^E/E^* (wt) or *Mc1r^e/e^ (extension)* background in which neither eumelanin nor pheomelanin can be made because of defective tyrosinase function (Jarrett et al., 2014). Note that these animals express the *K14-Scf* transgene resulting in retention of interfollicular epidermal melanocytes and pigmentation of the skin in addition to the fur.

Melanocortin 1 receptor is a highly polymorphic protein, and in humans many of the loss-of-function variants are associated with the “red hair color” (RHC) phenotype (Valverde et al., 1995; Box et al., 1997; Smith et al., 1998; Abdel-Malek et al., 2014). Individuals with a dysfunctional MC1R may have decreased eumelanin synthesis leading to fair skin and an increased sensitivity to UV exposure (Smith et al., 1998; Palmer et al., 2000; Landi et al., 2005). Degree of MC1R function correlates with the extent of pigmentation phenotype in individuals with RHC variants (Beaumont et al., 2007), and the effects of MC1R on basal pigmentation can be seen in both humans and murine models. Murine coat color is heavily influenced by MC1R signaling as clearly evident by variations in coat color associated with MC1R mutations such as the *extension* locus (**Figure 4**). Mice with the *recessive yellow* mutation (mutation of the *extension* locus) produce a non-functional MC1R and exhibit a blonde pheomelanotic coat color as a result (Robbins et al., 1993). Conversely, an increase in MC1R activity found in either the *somber* (constitutive active receptor) or *tobacco* (hyperactive receptor) mutation is associated with an increase in eumelanin synthesis and a darker coat color (Robbins et al., 1993) although no gain of function mutations have been identified in the human MC1R gene. The effect of MC1R signaling on basal pigmentation can be further seen in the *lethal yellow* mutation which affects an MC1R ligand rather than MC1R directly. Mice with the *lethal yellow* mutation have a blonde pheomelanotic coat color due to the ubiquitous overexpression of the negative agonist murine homolog of ASIP (ASP) inhibiting which diminishes basal Mc1r activity (Lovett et al., 1987).

#### Adaptive Tanning

The ability of the skin to respond to UV radiation by increasing melanin production is dependent on the functionality of MC1R. The adaptive pigmentation pathway represents a major innate protective mechanism by which the skin prevents further damage from ultraviolet radiation and is dependent upon MC1R signaling. UV radiation causes DNA damage to keratinocytes in the epidermis of the skin and the subsequent increased expression of the POMC protein in a p53 dependent manner (Cui et al., 2007). Cleavage of POMC by proconvertase 1 and 2 leads to the generation of the positive agonist α-MSH (Benjannet et al., 1991). Binding of α-MSH to MC1R leads to the activation of adenylyl cyclase and promotes the generation of cAMP (Kadekaro et al., 2003; Millington, 2006). cAMP accumulation promotes the activation of PKA leading to the phosphorylation of the cAMP responsive binding element (CREB). CREB functions as a transcription factor causing the upregulation of microphthalmia transcription factor (MITF). MITF functions as a master transcription factor and leads to the increased expression of multiple pigment dependent enzymes including tyrosinase (Levy et al., 2006).

#### MC1R and Pigment-independent UV Protection

Besides its role in regulating melanocyte pigment production, MC1R is a critical determinant of cellular genome maintenance pathways (Abdel-Malek et al., 2014). Melanocortins augment melanocyte anti-oxidant defense mechanisms and diminish free radical injury in melanocytes (Kokot et al., 2009; Song et al., 2009; Kadekaro et al., 2012). MC1R signaling also plays a critical role in the ability of melanocytes to recover from UV damage, particularly with respect to damage to genomic DNA. UV causes direct damage to pyrimidine bases within DNA that promote mutagenesis if not cleared from the genome. The NER pathway is the major means by which cells remove UV photoproducts from genomic DNA (reviewed in Scharer, 2013 and Shah and He, 2015) (Scharer, 2013; Shah and He, 2015).

##### Nucleotide excision repair

Nucleotide excision repair is the major genome maintenance pathway by which cells remove bulky DNA lesions that distort the DNA double helical structure including UV induced photoproducts (6,4 photoproducts and cyclobutane dimers). The NER pathway involves the coordinated action of multiple factors that recognize, excise, and repair damaged nucleotides. There are two types of NER - global genome nucleotide excision repair (GG-NER) and transcription coupled nucleotide excision repair (TC-NER). They differ in the initial stages of damage recognition, ultimately converging on a common repair pathway. TC-NER is invoked when transcription is stalled by nucleotide lesions in actively transcribed genes (Mellon et al., 1987; Mu and Sancar, 1997; Sugasawa et al., 1998, 2001). TC-NER is mediated by Cockayne syndrome B (CSB) and Cockayne syndrome A (CSA) proteins which are recruited to helical distortions that have caused RNA polymerase to stall (Mellon et al., 1987; Venema et al., 1991; Donahue et al., 1994; Mu and Sancar, 1997; Kamiuchi et al., 2002). In GG-NER, xeroderma pigmentosum complementation group C (XPC) and HR23B heterodimerize and scan the genome for helical distortions (Sugasawa et al., 1998, 2001). After damage recognition, TFIIH, a multiprotein complex composed of nine proteins, is recruited. TFIIH contains the helicases XPB and XPD which function in the 3′-5′ and 5′-3′ directions, respectively, (Gerard et al., 1991). The helicases unwind approximately 20–30 nucleotides surrounding the DNA lesion creating two unprotected single strand sequences. RPA and XPA are recruited to stabilize the open DNA conformation (Tapias et al., 2004; Park and Choi, 2006) followed by the endonucleases ERCC1-XPF and XPG which remove the damaged base (Mu and Sancar, 1997; Houtsmuller et al., 1999). Polymerase δ and 𝜀 in combination with proliferating cell nuclear antigen replace the gap using the undamaged complementary strand for fidelity (Shivji et al., 1995; Cleaver, 2005; Shah and He, 2015). In this way, NER efficiently repairs UV photoproducts and prevents UV mutagenesis.

##### MC1R signaling and NER

**FIGURE 5:**
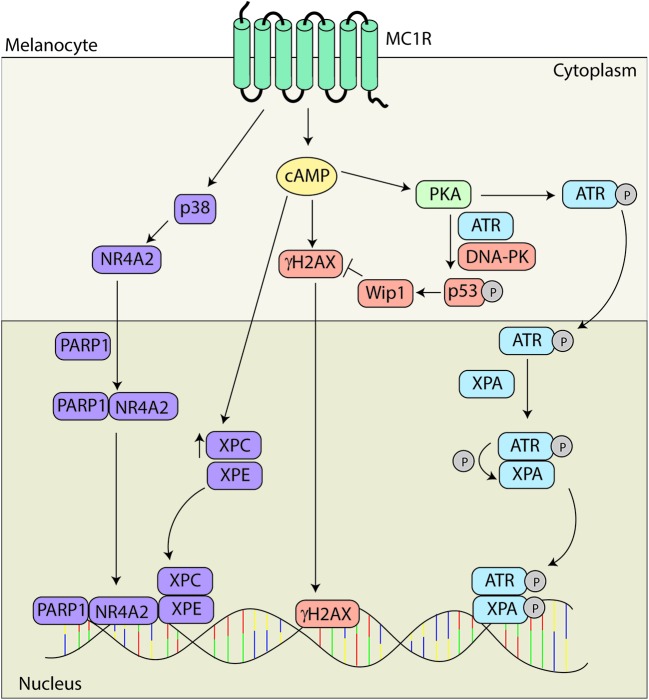
**Melanocortin 1 receptor signaling and melanocyte genomic integrity.** MC1R signaling promotes genomic stability through multiple mechanisms. MC1R activation induces translocation of NR4A2 to the nucleus in a p38 and PARP1 dependent manner where it co-localizes with XPC and XPE at sites of UV induced DNA damage. MC1R activation also leads to elevated levels of XPC and γH2Ax promoting the formation of DNA repair complexes. Levels of γH2AX are regulated by Wip1 downstream of ATR and DNA-PK mediated phosphorylation of p53 at S15. In addition, PKA activation promotes the phosphorylation of ATR at S435. pS435-ATR complexes with XPA in the nucleus. Following phosphorylation of XPA, the complex translocates to sites of UV induced DNA damage to accelerate and enhance nucleotide excision repair (NER).

Individuals with defective MC1R signaling are prone to UV induced skin cancer not only because they have decreased pigmentation but also because they have a blunted DNA repair response. We and others have reported that activation of MC1R and the subsequent cAMP signaling cascade are major regulators of NER kinetics and efficiency independent of pigmentation (**Figure 5**) (Kadekaro et al., 2005, 2010; Smith et al., 2008; Jagirdar et al., 2013; Jarrett et al., 2014; Swope et al., 2014). cAMP signaling directly impacts how long UV photodamage persists in melanocytes (Hauser et al., 2006; Abdel-Malek et al., 2009), and repair of photodamage in the skin of K14-Scf mice is much more robust when Mc1r is functional or when pharmacologic agents are topically applied to the skin that induce cAMP signaling (Jarrett et al., 2014).

Although it has been known for a decade that MC1R signaling accelerates NER kinetics (Abdel-Malek et al., 2006), the molecular mechanisms by which the phenomenon occurs have only recently begun to be elucidated and appear to be complex. Acceleration of repair of photo damage has been shown to be dependent upon both the nuclear receptor subfamily 4 group A member 2 (NR4A2) and ataxia telangiectasia mutated and Rad3 related (ATR) signaling pathways (Smith et al., 2008; Jagirdar et al., 2013; Jarrett et al., 2014). MC1R signaling leads to the induction of the NR4A2 which translocates to sites of photodamage in a p38 and poly ADP ribose polymerase (PARP) dependent manner. The NR4A2/PARP complex colocalizes with the DNA damage proteins XPC and XPE at sites of DNA damage. Data suggests that NRFA2 may play a role in promoting chromatin relaxation to promote DNA repair (Smith et al., 2008; Jagirdar et al., 2013).

Recently, we reported that enhancement of NER by cAMP is dependent on a post-translational modification of ATR protein. Specifically, we found that MC1R stimulation by melanocortins or pharmacological cAMP induction caused PKA to phosphorylate ATR at S435. This event promoted enhanced binding to the NER factor XPA in the nucleus and together pS435-ATR and XPA localized to UV photodamage in an accelerated and robust manner. Without PKA-mediated phosphorylation of ATR on S435, we observed no enhancement of NER in melanocytes. Therefore we concluded that MC1R signaling regulates genomic stability through this ATR-dependent mechanism (Jarrett et al., 2014). In subsequent work, we found that the MC1R agonists α-MSH or ACTH stimulated PKA-mediated ATR phosphorylation and NER whereas the MC1R negative agonist ASIP and the neutral antagonist βD3 inhibited the pathway, suggesting that melanocyte genomic stability and susceptibility to UV mutagenesis may be hormonally influenced (Jarrett et al., 2015).

Activation of MC1R has also been shown to facilitate repair via an increase in DNA damage response proteins. Treatment with α-MSH leads to an increase in XPC and γH2AX levels promoting formation of DNA repair complexes in primary human melanocytes (Swope et al., 2014). There is also a concomitant increase in DNA repair gene expression dependent upon MC1R signaling (Kadekaro et al., 2010). In addition, MC1R signaling also promotes the return to homeostasis via p53 signaling. MC1R activation promotes the phosphorylation of p53 at S15 in an ATR and DNA-PK dependent fashion leading to activation of wild-type p53 induced phosphatase 1 and degradation of γH2AX (Kadekaro et al., 2012; Swope et al., 2014).

## Hormonal Regulation of MC1R

**FIGURE 6:**
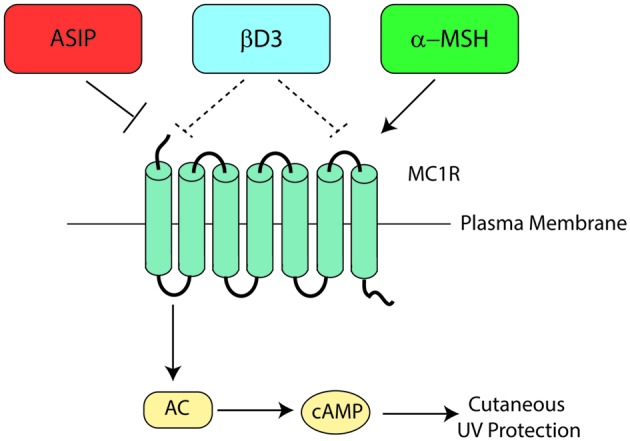
**Melanocortin 1 receptor ligand interactions.** Three major MC1R ligands are α-MSH, ASIP, and βD3. The melanocortin α-MSH functions as a positive agonist to increase cAMP levels downstream of MC1R. ASIP is a potent negative agonist that decreases basal MC1R signaling and interferes with melanocortin-induced cAMP upregulation. βD3 is thought to act as a neutral antagonist of MC1R, blocking the interactions with either melanocortins or ASIP but having little signaling impact independently. Each ligand appears to function as a competitive inhibitor for the others with only one able to bind to MC1R at any given time. Please note that although not shown in this diagram, ACTH’s ability to agonize MC1R is roughly the same as that of α-MSH.

Melanocortin 1 receptor signaling is complex and dynamic, with signaling heavily influenced by receptor interactions with melanocortins, agouti signaling protein (ASIP), or β-defensin 3 (βD3). Melanocortins enhance MC1R signaling, ASIP inhibits MC1R signaling directly, and the neutral antagonist βD3 blunts melanocortin-induced signaling by competing with MC1R agonists for MC1R binding (**Figure 6**). The major melanocortin for MC1R, α-MSH, functions to increase cAMP levels after binding to MC1R whereas binding of ASIP to MC1R competes with α-MSH binding to prevent melanocortin activation and results in a decrease in basal cAMP levels. Binding of βD3 to MC1R does not affect basal cAMP levels, however, it functions as a competitive inhibitor and interferes with binding of either α-MSH or ASIP.

### Melanocortins

**FIGURE 7:**
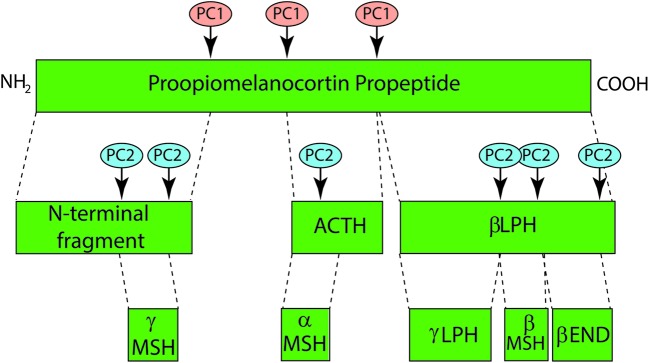
**Melanocortin synthesis through proopiomelanocortin (POMC) processing.** Melanocortins are derived from the proopiomelanocortin (POMC) precursor peptide that is cleaved into functional peptide fragments by a pair of serine protease pro-protein convertases, PC1 and PC2. PC1 cleaves POMC into four subunits including an n-terminal region from which γ-MSH is derived, adrenocorticotropic hormone (ACTH) and β-lipotropin (β-LPH) from which γ-LPH, β-MSH and β-endorphin are generated by PC2 cleavage. α-MSH represents the first 13 amino acids of ACTH and is dependent on PC2 activity for its synthesis.

There are four endogenous melanocortin ligands: α-MSH, β-MSH, γ-MSH, and adrenocorticotropic hormone (ACTH). Melanocortins are derived as cleavage products of the pro-opiomelanocortin (POMC) protein (Wintzen and Gilchrest, 1996) (**Figure 7**), and each has varying receptor binding affinities across the MC family. The *POMC* gene contains three exons and two introns with the bioactive peptides present in exon 3 (Wintzen and Gilchrest, 1996). POMC is processed by proconvertase 1 to generate ACTH, the N-terminal fragment, and beta-lipotropin (β-LPH). Further cleavage of ACTH by proconvertase 2 generates corticotropin-like intermediate peptide (CLIP) and α-MSH. Cleavage of the N-terminal fragment by proconvertase 2 generates γ-MSH. Cleavage of β-LPH generates γ-LPH and β-endorphin (Wintzen and Gilchrest, 1996) which is active at opiate receptors in the skin. The UV-dependent β-endorphin production and the resultant opiate “high” is believed to contribute to UV-seeking behavior and an increase in analgesic threshold due to opioid dependence (Fell et al., 2014). POMC expression and processing is induced in both keratinocytes and melanocytes following UV exposure, leading to the secretion of MC1R ligands α-MSH and ACTH (Schauer et al., 1994; Chakraborty et al., 1996) in a p53 dependent manner (Cui et al., 2007).

ACTH and α-MSH are the two major melanocortins for MC1R (Abdel-Malek et al., 2000). ACTH was first identified in Smith (1930), and α-MSH (first termed simply MSH prior to the discovery of β- and γ-MSH) was identified in the porcine pituitary Lerner and Lee (1955). α-MSH is expressed in both human melanocytes and keratinocytes (Chakraborty et al., 1996), and was first shown to bind to murine and human melanoma cells via a high affinity receptor Siegrist et al. (1989) and Solca et al. (1989). α-MSH binding to normal human melanocytes was demonstrated Donatien et al. (1992) and De Luca et al. (1993) by two independent investigators.

All melanocortin ligands have an HFRW motif required for receptor binding that contributes to the generalized ability for melanocortins to interact with more than one melanocortin receptor, albeit with different affinities. Binding to MC2R, however, also requires an additional motif, KKRRP, which is only present in ACTH. Therefore only ACTH can bind to MC2R (Mountjoy et al., 1992; Dores, 2009). Both MC1R (Abdel-Malek et al., 2000; Sanchez-Mas et al., 2004) and MC4R (Gantz et al., 1993) bind ACTH and α-MSH with similar affinities and each binds β- and γ-MSH with lower affinity. Similarly, MC5R binds α-MSH with the highest affinity followed by β-MSH, ACTH, and γ-MSH in order of decreasing affinity (Gantz et al., 1994a). Agonists interact with extracellular loops of melanocortin receptors as well as charged and aromatic residues in the TM fragments (Garcia-Borron et al., 2005). Specifically, the arginine of the melanocortin HFRW pharmacophore core interacts with E94 (TM2), D117, and D121 (TM3). Similarly, aromatic residues near the extracellular side of melanocortin receptor TM regions 4, 5, and 6 interact with aromatic residues of the melanocortin pharmacophore (Yang et al., 1997).

### Agouti Signaling Protein

The human agouti signaling protein is a 132 amino acid MC1R ligand encoded by the *agouti* locus on chromosome 20q11.2 with 85% homology to the murine protein (Miller et al., 1993; Kwon et al., 1994; Wilson et al., 1995). ASIP functions as a competitive MC1R inhibitor, efficiently preventing α-MSH binding to MC1R and inhibiting MC1R activation (Blanchard et al., 1995; Suzuki et al., 1997). Binding of ASIP and α-MSH to MC1R are mutually exclusive (Ollmann et al., 1998). In addition, ASIP functions as an inverse agonist to decrease basal MC1R signaling and inhibit eumelanogenesis (Lu et al., 1994; Wilson et al., 1995). Although ASIP’s primary sequence has no similarities to either ACTH or α-MSH, it binds to MC1R with almost equal affinity (Siegrist et al., 1996). ASIP’s cysteine rich C-terminal region binds to MC1R via an octaloop structure with four residues that are homologous to other melanocortin ligands (Ollmann et al., 1998; Tota et al., 1999; McNulty et al., 2005). The C-terminal region of ASIP is both necessary and sufficient for its effect at MC1R, and the C-terminus alone can function as a competitive antagonist to melanocortins at melanocortin receptors (Ollmann and Barsh, 1999).

Agouti signaling protein was known to promote a pheomelanotic coat phenotype before it was determined to be a direct ligand of MC1R. It is expressed in the dermal papilla of the hair follicle where it functions as a paracrine signal to regulate hair color (Millar et al., 1995). In the fur of a variety of animals, the *agouti* locus is expressed transiently to create alternating bands of pheomelanin and eumelanin on the hair shaft which results in improved camouflage (Tamate and Takeuchi, 1984). Canonically, ASP (the murine homolog of the human ASIP) is secreted locally only at the hair follicle, however, there are multiple murine models with varying coat phenotypes dependent upon altered agouti expression. For example, mice with the *lethal yellow* mutation (*Ay*), a dominant gain of function mutation resulting in ectopic and ubiquitous ASP expression, have a yellow/blonde coat color and are obese with hyperinsulinemia due to binding of the overexpressed ASP to MC4R (Takeuchi et al., 1989; Bultman et al., 1992; Robbins et al., 1993; Duhl et al., 1994a,b; Michaud et al., 1994).

The ability of ASIP to promote a pheomelanotic phenotype is dependent upon a functional MC1R, and ASIP had no effect on MC1R with constitutive activity or loss of function (Ollmann et al., 1998; Abdel-Malek et al., 2001). Treatment with ASIP inhibits α-MSH binding to MC1R preventing α-MSH induced cAMP production and tyrosinase, tyrosine related proteins 1 and 2, and MITF expression suppressing melanogenesis (Aberdam et al., 1998). ASIP’s inhibitory effect on pigment production, however, is seen with or without concomitant stimulation with α-MSH suggesting that the effects of ASIP at MC1R are not completely explained via preventing α-MSH binding (Graham et al., 1997). Treatment of primary murine or human melanocytes or melanoma cell lines with ASIP caused a decrease in eumelanosomes and an inhibition of pigmentation (Hunt and Thody, 1995; Sakai et al., 1997), and binding of ASIP to wild-type MC1R leads to decreased basal tyrosinase activity and decreased tyrosinase and tyrosinase related protein 1, 2, and 3 levels preventing eumelanogenesis (Sakai et al., 1997; Abdel-Malek et al., 2001).

Treatment with agouti also affects additional MC1R signaling pathways including NER, proliferation, and migration. Concomitant stimulation with ASIP and α-MSH abrogated α-MSH’s acceleration of NER following UV treatment (Jarrett et al., 2014, 2015), decreased basal DNA repair kinetics in a dose dependent manner and decreased basal ATR phosphorylation at S435 in primary melanocytes (Jarrett et al., 2015). Consistent with inhibition of MC1R signaling, treatment with ASIP leads to a decrease in proliferation in a mouse melanoma cell line (Siegrist et al., 1996) and an increase in melanocyte migration (Le Pape et al., 2009).

ASP functions with two major accessory proteins: attractin and mahogunin. Attractin is encoded by the *Atrn* gene and is a large single transmembrane protein (Gunn et al., 1999; Nagle et al., 1999) with an ectodomain that binds the amino terminus of ASP (He et al., 2001) and is absolutely required for ASP signaling *in vivo*. Although it is unknown whether attractin, ASP, and Mc1r form a complex, mice without a functional attractin protein are unresponsive to ASP signaling suggesting attractin is required for ASP function at Mc1r. Mahogunin is encoded by the *Mgrn1* gene and is an intracellular protein that functions as an E3 ubiquitin ligase (Phan et al., 2002; He et al., 2003a,b).

Mutations in murine *Atrn* or *Mgrn1* (formerly the *mahogany* and *mahoganoid* mutations, respectively) are associated with eumelanotic phenotypes similar to that seen in either a mutation causing loss of function *Agouti* mutations or a gain of function *Mc1r* mutation (Lane and Green, 1960). Mutations in *Atrn* or *Mgrn1* have no effect on plasma α-MSH or ACTH levels, however, they do interfere with agouti signaling and prevent it from binding to MC1R effectively thereby resulting in a darkened coat color (Miller et al., 1997). The interaction between attractin, mahogunin, and ASP are unclear, however, a recent study suggests that attractin and mahogunin mediated ASIP signaling is cAMP independent, however, the cAMP dependent ASIP signaling requires neither attractin nor mahogunin (Hida et al., 2009).

### Beta Defensin 3 (βD3)

The defensins are a group of antimicrobial peptides that link innate and adaptive immune responses. They are small cationic amphiphilic proteins composed of 30–40 amino acids (Boman, 1995; McCray and Bentley, 1997; De Lucca and Walsh, 1999; Schneider et al., 2005) (Kesting et al., 2010) that target gram positive and gram negative bacteria in addition to fungi (Garcia et al., 2001; Schneider et al., 2005; Arnett and Seveau, 2011) by binding to negatively charged membrane components (Schneider et al., 2005). Although there are two major classes of defensins, α and β (Schneider et al., 2005), only the β-defensins have been identified in the skin (Bensch et al., 1995; Weinberg et al., 1998; Huttner and Bevins, 1999; Schroder and Harder, 1999). Although βD1, βD2, and βD3 are all expressed in the skin, βD3 appears to be unique in its ability to bind the MC1R and influence melanocyte physiology (e.g., mammalian coat color). βD3 is synthesized by keratinocytes in the spinous and granular layers (Sawamura et al., 2005) and functions in a paracrine manner by binding to MC1R on interdigitating melanocytes (Harder et al., 2001; Garcia-Borron et al., 2005; Candille et al., 2007) via electrostatic interactions (Nix et al., 2013).

Clarence Cook Little was the first to demonstrate that the dominant inheritance of black coat color in dogs was independent of MC1R mutations and suggested that the color was due to an unusual allele of agouti (Little, 1957; Candille et al., 2007). However, Candille et al. (2007) identified the locus responsible for Little’s observation as CBD103 (β defensin 103), the canine homolog of βD3, rather than an agouti variant. Indeed, overexpression of β defensin 103 and its subsequent binding to MC1R inhibited interactions between ASIP and MC1R, preventing the dogs from developing a blonde coat (Candille et al., 2007). The ability of βD3 homologs to promote mammalian black coat color requires a functional MC1R protein, showing β defensin 103’s effects are mediated by interactions with MC1R rather than other melanocyte surface receptors (Nix et al., 2013).

Although regulation of βD3 following inflammatory stimuli is established, its regulation in keratinocytes following exposure to UV radiation is not well-characterized. βD3 expression is induced in keratinocytes in the setting of inflammation or wound formation (Kesting et al., 2010) and is mediated by inflammatory cytokines including TNFα, IL-7, and IL-1β (Harder et al., 2001). In contrast, neither TNFα nor IL-17 influence POMC or ASIP expression, suggesting βD3 may be independently regulated compared to the other melanocortins and that inflammatory-mediated pigment changes may be βD3-dependent (Wang et al., 2013). UVB radiation, which induces POMC gene expression both *in vivo* and *in vitro*, has variable effects on βD3 induction, depending upon the experimental model. *In vivo* exposure of adult skin to UVB radiation led to increases in βD3 gene and protein expression (Glaser et al., 2009), however, UV exposure of *ex vivo* neonatal skin explants did not induce βD3 expression suggesting that βD3 up-regulation following UVB exposure may require recruitment of additional cell types, potentially cytokine-producing immune cells, to the skin (Wolf Horrell and D’Orazio, 2014).

Multiple reports have demonstrated that βD3 binds MC1R. In MC1R-transfected human embryonic kidney cells (HEK293), βD3 functioned as a weak agonist and promoted cAMP accumulation and MAP kinase activation (Beaumont et al., 2012). However, in MC1R^wt^ primary melanocytes or in human melan-a melanocytes, βD3 acted as a neutral MC1R antagonist, exerting little influence on cellular cAMP levels independently but preventing MSH and ASIP binding to MC1R (Swope et al., 2012; Nix et al., 2013). Similarly, Candille et al. (2007) found evidence that the canine homolog CBD103 functioned as a neutral MC1R antagonist. Finally, we found that βD3 inhibited α-MSH-induced generation of pS435-ATR and enhancement of DNA repair (Jarrett et al., 2015). Thus most available data support the hypothesis that βD3 acts as a neutral MC1R antagonist. Importantly, regardless of experimental system, βD3 is unable prevent the induction of cAMP and downstream melanocyte differentiation pathways (e.g., pigment induction) by the direct adenylyl cyclase activating drug forskolin confirming that βD3 functions at the level of the MC1R rather than by inhibiting the adenylyl cyclase enzyme or other downstream MC1R signaling event (Swope et al., 2012).

## Targeting the Melanocortin-MC1R Axis

The *MC1R* gene is a highly polymorphic genetic locus and inherited defects in MC1R function are common among fair-skinned, UV-sensitive and melanoma-prone persons. Indeed, there may be up to 6–8 million Americans harboring double allele polymorphisms and millions more being hemizygous for MC1R (Kennedy et al., 2001). Individuals with defective MC1R signaling are prone to melanoma and other UV induced skin cancers not only because their skin is under-melanized but also because they have a blunted melanocytic DNA repair response. Because MC1R-mediated UV protection and melanoma resistance is proportional to the robustness of the cAMP response downstream of MC1R signaling and a variety of pharmacologic strategies exist to impact cAMP, it might be possible to exploit MC1R signaling as a UV- and melanoma-preventive strategy. For example, topical application of either forskolin, a direct activator of adenylyl cyclase, or rolipram, a phosphodiesterase inhibitor, potently rescued eumelanin production in pheomelanotic *Mc1r*-defective C57BL/6 animals and protected the skin against UV damage (D’Orazio et al., 2006; Khaled et al., 2010). Similarly, topical forskolin promoted clearance of UV photoproducts in the skin (Jarrett et al., 2014). These proof-of-principle studies indicate that beneficial effects of MC1R signaling can be induced by pharmacologic manipulation cAMP levels in the skin. Topical application of agents that induce cAMP production or prevent its clearance, while effective, lacks melanocyte specificity and off-target effects must be considered before this approach can be deemed safe or appropriate for translational use. Melanocortin analogs under development offer a more targeted approach (Abdel-Malek et al., 2006), but their efficacy is dependent on expression of functional MC1R and therefore individuals with homozygous or compound heterozygous MC1R defects may not benefit from these agents. Although there is clearly a need for much more investigation into the mechanisms, feasibility and consequences of pharmacologic MC1R targeting, the melanocortin-MC1R signaling axis may prove to be a useful target for rational development of novel UV-resistance and melanoma-preventive strategies.

### Therapeutic Implications and Perspectives

Taking into account the abundance of data linking MC1R function to the vigor of melanocytic UV physiologic responses, it is clear that the MSH-MC1R signaling axis represents a critical innate UV-protective mechanism in the skin. Whereas its importance was first appreciated based on its contribution to melanin biosynthesis, more recent studies have documented a direct link between cAMP signaling and melanocyte genomic stability. It is now well-established that melanoma ranks among the highest of human tumors with respect to somatic mutational load (Hodis et al., 2012; Lawrence et al., 2014) and that UV signature mutations account for the majority of melanoma-associated mutations (Shain et al., 2015). Epidermal melanocytes, the precursor cells that give rise to melanoma, are long-lived cells that because of their position in the skin, must cope with intermittent UV damage. In addition to overall UV dose and the pattern of UV injury (acute vs. chronic), it is likely that UV mutagenesis (and ultimately melanoma incidence) will be heavily influenced by the efficiency by which melanocytes repair nuclear UV photodamage. MC1R-defective individuals, because of their tendency to be undermelanized (which allows more UV to penetrate into the basal layer of the epidermis) and because they lack the DNA repair “boost” that an effective MSH-MC1R axis yields, should accumulate more UV mutagenesis over time and would therefore be at higher risk for melanoma as a result. Pharmacologic “rescue” of cAMP signaling would protect such individuals by enhancing pigment production and by promoting genomic stability. Intriguingly, MC1R function might also predict therapeutic response to immune-based anti-melanoma therapies. Though this has yet to be formally tested, it is possible that melanomas with higher somatic mutational burdens (as might occur in the MC1R-defective state) might respond better to immune checkpoint blockade because they would express more mutated proteins, any one of which might be recognized as a tumor-associated antigen for cytotoxic lymphocytes. Because of its central regulatory role for a host of melanocyte physiological responses, the MC1R signaling pathway is emerging as an ever-increasingly important pharmacologic target in preventing or treating melanoma.

## Author Contributions

EMW and MCB wrote the review article along with JAD who helped write and edit the manuscript and figures.

## Conflict of Interest Statement

The authors declare that the research was conducted in the absence of any commercial or financial relationships that could be construed as a potential conflict of interest.
